# Literary Notes

**Published:** 1900-07

**Authors:** 


### LITERARY NOTES.

The *Journal of Surgical Technology* is the title of a new periodical to be published monthly, beginning July 1, 1900. It will be devoted to the consideration of the technic of surgical procedures, at a subscription price of $1.00 a year. Premiums are offered with the first subscriptions. Address the Technique Publishing Co., 404 East 14th street, New York City, N. Y.

THE *Memphis Medical Monthly* one of our sterling exchanges, has absorbed the *Memphis Lancet*, a most excellent step in the direction of consolidation and reduction of the medical journal force.

---

MESSRS. WILLIAM WOOD & CO., medical publishers of New York, announce the early republication of the Reference Handbook of the Medical Sciences, edited by Dr. Albert H. Buck of New York City. When this work was first issued it proved to be very popular and its success was such that it inaugurated an entirely new system in the production of encyclopedic medical works.

The rapid advances in medicine and surgery during recent years necessitates entirely rewriting the work from beginning to end. The staff of department advisers and contributors, numbering in all about 500 physicians, are at work upon it and it is expected the first volume will be issued about August 15, 1900.

The illustrations will be of the finest quality including many exquisite chromo-lithographic plates. The paper will be highly finished and altogether it will be a superb edition.

